# Lack of TRPV1 Channel Modulates Mouse Gene Expression and Liver Proteome with Glucose Metabolism Changes

**DOI:** 10.3390/ijms23137014

**Published:** 2022-06-24

**Authors:** José Thalles Lacerda, Patrícia R. L. Gomes, Giovanna Zanetti, Nathana Mezzalira, Otoniel G. Lima, Leonardo V. M. de Assis, Ali Guler, Ana Maria Castrucci, Maria Nathália Moraes

**Affiliations:** 1 Laboratory of Comparative Physiology of Pigmentation, Department of Physiology, Institute of Biosciences, University of São Paulo, São Paulo 05508-090, Brazil; thalles_lacerda2@hotmail.com (J.T.L.); giovannazanetti@usp.br (G.Z.); nathana.mezzalira@unifesp.br (N.M.); otonielglima@ib.usp.br (O.G.L.); deassis.leonardo@alumni.usp.br (L.V.M.d.A.); amdlcast@ib.usp.br (A.M.C.); 2 Laboratory of Neurobiology, Department of Physiology and Biophysics, Institute of Biomedical Sciences, University of São Paulo, São Paulo 05508-000, Brazil; rlg.paty@gmail.com; 3 Institute of Neurobiology, University of Lübeck, 23562 Lübeck, Germany; 4 Department of Biology, University of Virginia, Charlottesville, VA 22904, USA; aguler@virginia.edu

**Keywords:** glycogenolysis, gluconeogenesis, liver clock and glucose metabolism genes, proteomics

## Abstract

To investigate the role of the transient receptor potential channel vanilloid type 1 (TRPV1) in hepatic glucose metabolism, we analyzed genes related to the clock system and glucose/lipid metabolism and performed glycogen measurements at ZT8 and ZT20 in the liver of C57Bl/6J (WT) and *Trpv1* KO mice. To identify molecular clues associated with metabolic changes, we performed proteomics analysis at ZT8. Liver from *Trpv1* KO mice exhibited reduced *Per1* expression and increased *Pparα*, *Pparγ*, *Glut2*, *G6pc1* (*G6pase)*, *Pck1* (*Pepck*), *Akt*, and *Gsk3b* expression at ZT8. Liver from *Trpv1* KO mice also showed reduced glycogen storage at ZT8 but not at ZT20 and significant proteomics changes consistent with enhanced glycogenolysis, as well as increased gluconeogenesis and inflammatory features. The network propagation approach evidenced that the TRPV1 channel is an intrinsic component of the glucagon signaling pathway, and its loss seems to be associated with increased gluconeogenesis through PKA signaling. In this sense, the differentially identified kinases and phosphatases in WT and *Trpv1* KO liver proteomes show that the PP2A phosphatase complex and PKA may be major players in glycogenolysis in *Trpv1* KO mice.

## 1. Introduction

The transient receptor potential channel vanilloid type 1 (TRPV1) is a member of the TRP channel superfamily, comprised of a large group of nonselective cation channels activated by heat, protons, capsaicin, and others [1]. They are expressed in many tissues and display a wide variety of physiological functions, including metabolic regulation [1,2,3]. TRP channels have been shown to be involved in the control of appetite and body weight [4,5], pancreatic function [6], glucose metabolism [7,8,9], and the development and progression of diabetes mellitus [10]. In this sense, mice lacking TRPV1 channels exhibit impairment in glucose metabolism, especially evidenced by reduced glucose-induced insulin secretion and high blood glucose levels after the glucose tolerance test [8]. On the other hand, TRPV1 activation by capsaicin in C2C12 cells leads to the recruitment of CAMKK2 and AMPK signaling pathways, which results in increased glucose oxidation and ATP production in an insulin-independent way [9] and increased hepatic glycogen storage in streptozotocin-induced diabetic rats [11]. Pharmacological inhibition of TRPV1 results in decreased CAMKK2 and AMPK activation and, thus, reduced glucose oxidation and ATP production [9]. AMPK is an internal energy sensor that responds to decreased nutrient and energy levels by activating calcium signaling to restore energy levels. Interestingly, the absence of TRPV channels blocks glucose-starvation-induced AMPK activation in the liver [12].

In addition to their metabolic function, TRP channels have been reported to be involved in the regulation of clock genes [13,14]. Our group has demonstrated that the clock molecular machinery of brown adipose tissue (BAT) is affected in the absence of TRP channel types V1 [14] and M8 [13]. Interestingly, in ZEM-2S cells of the teleost *Danio rerio*, we found that heat shock-induced *per2* increase is partially dependent on TRPV1 [15].

The liver performs several functions in different phases of the circadian clock, which is mainly modulated by transcriptional programs [16] and post-translational regulation [17]. During physiological fasting (ZT0 to ZT12), liver metabolism is directed to glycogenolysis, gluconeogenesis, amino acid catabolism, and fatty acid oxidation, and during feeding (ZT12 to ZT24), it switches to glycogenesis, glycolysis, amino acid anabolism, and lipogenesis [18]. The mechanism of glucose regulation involves a variety of liver genes that exhibit robust circadian regulation, suggesting that the circadian clock plays a role in hepatic glucose metabolism [19,20,21]. The activities of the rate-limiting enzymes for glycogenesis and glycogenolysis also show circadian rhythms, and the balance between them contributes to the circadian rhythm of hepatic glycogen content. Accordingly, *Clock* KO [22] or *Bmal1* KO mice [18] display reduced mRNA and protein expression of GYS2 (glycogen synthase 2), which is the rate-limiting enzyme of glycogenesis in the liver. Analysis of the hepatic transcriptome and proteome has shown circadian variation in the expression of many genes and proteins related to energy metabolism, including those regulating gluconeogenesis, lipogenesis, mitochondrial biogenesis, and oxidative metabolism, suggesting that liver circadian clocks are involved in these processes [16,20].

Having this in mind, we hypothesized that hepatic glycogen storage could be compromised in the absence of TRPV1 channels and that this metabolic alteration could be mediated by altered signaling pathways involving genes and proteins related to glucose homeostasis. In fact, we demonstrate that the TRPV1 channel contributes to the regulation of clock genes and genes involved in hepatic metabolism. The TRPV1 channel plays a critical role in the modulation of glycogenolysis and gluconeogenesis. Proteomic analyses indicate that the absence of TRPV1 channels may induce inflammatory features related to oxidative stress and increased gluconeogenesis. We also show that the TRPV1 channel is directly associated with glucagon signaling during the fasting period and with changes in kinases and phosphatases involved in glycogen breakdown. Therefore, we demonstrate that the TRPV1 channel is an important regulator of the liver molecular clock and glucose physiological processes.

## 2. Results and Discussion

### 2.1. Clock and Metabolic Hepatic Genes Are Altered in the Absence of TRPV1 Channels

**Figure 1:**
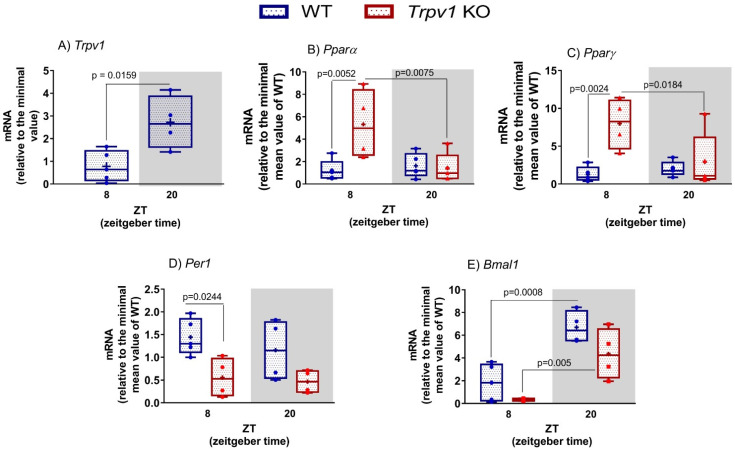
Gene expression in the liver of C57BL/6J WT and *Trpv1* KO mice. (**A**–**E**) Gene expression in the liver at ZT8 (8 h after lights on) and ZT20 (8 h after lights off). Results are shown as median, quantiles, and maximum and minimum expression values of genes of interest normalized by 18S ribosomal RNA and expressed relative to the minimal value of WT mice. + represents the mean (*n* = 4–5). In (**A**), statistical analysis was performed by Student’s *t*-test. (**B**–**E**) Significant differences between ZTs and between genotypes were demonstrated by two-Way ANOVA, followed by the Bonferroni post-test.

The expression of some TRP channels has been demonstrated in the liver [23]; however, their function has yet to be revealed. Hence, we analyzed *Trpv1* expression in WT mice at ZT8 and ZT20, and we found a lower expression of *Trpv1* at ZT8 compared to ZT20 (*p* = 0.0159, Figure 1A). Since TRP channels are involved in energy metabolism [1,7] and we have demonstrated that *Tprv1* displays a significant difference in its expression at ZT8 and ZT20, one could speculate that TRPV1 channels are involved in the regulation of hepatic functions. To the best of our knowledge, a functional analysis of calcium influx via TRPV1 in a time-dependent manner has yet to be demonstrated.

One of the most evident rhythms observed in almost all animals is the fasting/feeding cycle in association with the sleep/wake cycle [24,25]. This feature enhances energetic efficiency through the temporal segregation of anabolic and catabolic reactions such as gluconeogenesis and glycolysis. An important player that links metabolism regulation and circadian rhythm is a member of the peroxisome proliferator-activated receptor (PPAR) family that functions as a transcription factor [26,27]. In addition to this and having in mind that the TRPV1 channel is involved in glucose homeostasis [8], we started evaluating the daily expression of *Pparα* and *Pparγ* in the liver of mice lacking the TRPV1 channel. Statistical analysis performed by two-way ANOVA pointed to a significant difference in the expression of *Pparα* related to genotype, time, and interaction of these factors (Table 1). We found that only *Trpv1* KO mice displayed daily variation in the *Pparα* transcript (*p* = 0.0075) when ZT8 was compared to ZT20 (Figure 1B). Increased *Pparα* expression at ZT8 was observed in the absence of TRPV1 channels in comparison to WT at the same time point (*p* = 0.0052, Figure 1B). A similar profile was observed in the expression of *Pparγ* (Figure 1C); F analysis indicated that genotype and interaction of genotype with time are factors that contribute to the difference observed in the groups (Table 1). Expression of *Pparγ* did not vary between ZT8 and ZT20 in the liver of WT mice, while it did in the *Trpv1* KO liver (*p* = 0.0184, Figure 1C). Even though liver *Pparα* and *Pparγ* from WT did not show temporal variation between ZT8 and ZT20, it is well described that these genes display circadian variation, with a peak at the transition between light and dark phases and in the middle of the light phase, respectively [28,29].

PPARs, which act as transcription factors, regulate the expression of a variety of target genes, especially those related to energy metabolism [30]. It has long been known that metabolism and clock genes are tightly intertwined and that the PPAR family contributes to this process [26]. PPAR*α* and PPAR*γ* are direct regulators of the core clock components *Bmal1* and *Rev-erb*α**, and, conversely, PPAR*α* is also a direct *Bmal1* target gene [26]. In addition, we have previously demonstrated that in the absence of TRPV1 channels, clock genes are downregulated in brown adipose tissue (BAT) [14]. Hence, our next step was to investigate the contribution of TRPV1 channels to the regulation of hepatic clock genes. Analysis of the F test indicated genotype as the only factor that showed statistical significance (Table 1). At ZT8, we found reduced *Per1* expression in the absence of TRPV1 channels when compared to WT mice (*p* = 0.0244, Figure 1D). It is known that clock genes oscillate in the liver; however, the comparison between ZT8 and 20 in both WT and *Trpv1* KO mice was not able to provide such information (Figure 1D), which could be due to the time points analyzed here. In the liver of both WT and *Trpv1* KO mice, two-way ANOVA indicated significant differences in *Bmal1* expression related to genotype and time but not in the interaction of these two factors (Table 1). In this sense, we observed a daily variation of *Bmal1* mRNA in both genotypes (Table 1) when ZT8 was compared to ZT20 (WT *p* = 0.0008 and *Trpv1* KO *p* = 0.0055). Although the F test points to genotype as a significant factor, no difference in *Bmal1* expression was seen in the absence of TRPV1 channels when the genotypes were compared at the same time points (Figure 1E). The antiphase relationship between *Per1* and *Bmal1*, a known feature of synchronized molecular machinery, was observed in both genotypes. In summary, the lack of TRPV1 channels led to the reduced expression of clock genes, at least *Per1* and *Bmal1*, but this reduction was not related to the oscillatory expression profile (day–night variation) of these genes. In BAT, the involvement of TRPV1 and TRPM8 channels with temporal control of the local clock machinery is clear since the expression of clock genes was drastically reduced in *Trpv1* and *Trpm8* KO mice compared to WT mice [13,14].

Liver circadian gene oscillation is evidenced by the study of Zhang and colleagues [31], who demonstrated that among the 19,788 genes analyzed, 16% showed a circadian variation. It is well known that the circadian system regulates, at gene and protein levels, a series of enzymes involved in glucose and lipid metabolism [32]. Temporal oscillation in triglyceride and cholesterol levels in plasma is known to be high during the feeding period, while triglyceride breakdown and free fatty acid mobilization in the adipose tissue are maintained during the fasting period [33,34].

In addition to the involvement of the TRPV1 channel in the regulation of liver clock genes in our experimental setting, its role in metabolic functions has become an attractive research theme. In fact, *Trpv1* KO mice are more insulin resistant after consuming a high-fat diet (HFD) in comparison to HFD-fed WT mice [5]. Following this line, the activation of TRPV1 channels by a diet supplemented with capsaicin, a TRPV1 channel agonist, was shown to decrease lipid accumulation and triglyceride levels in the liver. In addition, TRPV1 activation increased the expression of UCP2, which plays a role in hepatic lipid metabolism and mitochondrial bioenergetics [35]. In accordance with these results, *Trpv1* KO [5] and *Trpv2* KO mice [36] are more susceptible to HFD-induced weight gain than WT mice and show an increased mass of fat deposits.

**Figure 2:**
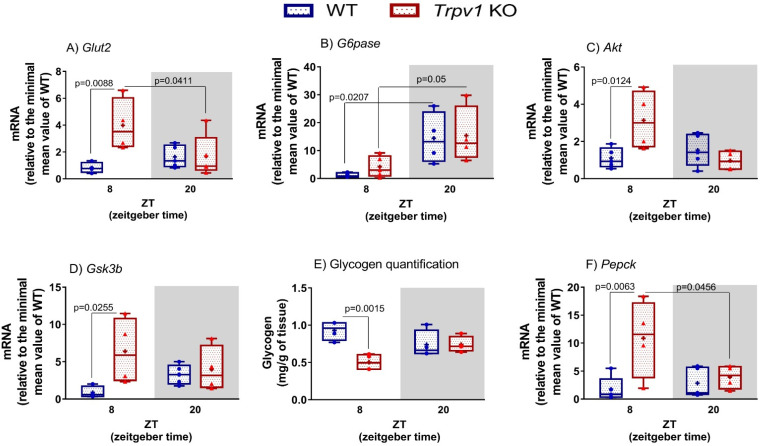
Hepatic glucose metabolism in the absence of TRPV1 channels. (**A**–**D**,**F**) Relative transcripts of hepatic genes related to glucose metabolism in C57BL/6J WT and *Trpv1* KO mice at ZT8 (physiological fasting) and ZT20 (eating phase). Results of mRNA quantification are shown as median, quantiles, and maximum and minimum expression values of gene transcripts normalized by 18S ribosomal RNA and expressed relative to the minimum mean value of WT mice. ₊ represents the mean (*n* = 4–5). Significant differences between ZTs and between genotypes were demonstrated by two-way ANOVA, followed by the Bonferroni post-test. (**E**) Hepatic glycogen quantification is expressed as mass (mg/g of tissue).

The maintenance of blood glucose homeostasis represents one of the fundamental challenges in the regulation of body metabolism, and circadian control has an important role in this scenario [37]. In this sense, it is known that glucose levels and tolerance and insulin action are known to vary throughout the day [24]. Considering that clock and PPAR genes are altered in the absence of TRPV1 channels, we decided to investigate how the TRPV1 channel participates in the regulation of hepatic glucose metabolism genes. One of the important functions of the liver is glucose homeostasis, and GLUT2 is the prevalent glucose transporter in this tissue [38]. We did not find temporal differences in the expression of *Glut2* in the liver of WT mice, at least at the time points analyzed here. On the other hand, in the absence of TRPV1 channels, an increase in *Glut2* expression was observed at ZT8 compared to WT mice. The increased expression of *Glut2* in the light phase resulted in a daytime variation in its transcript (Figure 2A). In fact, the F test points out that genotype and the interaction between time and genotype are significant factors for *Glut2* transcript regulation (Table 1).

We subsequently evaluated how the lack of TRPV1 would affect liver physiology by assessing the gene expression of *G6pase*, *Akt,* and *Gsk3β* in mice of both genotypes. In agreement with the literature reports on C57BL/6J mice [22,39], *G6pase* mRNA was found to vary in the liver of WT and *Trpv1* KO mice. The highest expression was identified in the dark phase (ZT20) compared to the light phase (ZT8, Table 1 and Figure 2B). No differences were found between genotypes, as demonstrated by the F test (Table 1). Upon insulin interaction with its receptor, the phosphoinositide 3-kinase (PI3K)–AKT pathway is activated, resulting in AKT phosphorylation. Active AKT signals to multiple downstream pathways to control, among other functions, glycogen synthesis through the inhibition of GSK3β [40]. Intriguingly, the expression of both *Akt* and *Gsk3β* was increased at ZT8 in the absence of TRPV1 channels (Figure 2C,D). No temporal variation was seen for both genes (Table 1).

Keeping in mind that genes involved in hepatic glucose homeostasis showed increased transcripts in the fasting phase (ZT8), mainly because we found high expression of *Glut2* in the liver of *Trpv1* KO mice, we questioned whether hepatic glycogen storage would be altered in the absence of the channel. We found a lower glycogen concentration in the liver of *Trpv1* KO mice, only at ZT8, compared to WT mice (Figure 2E). Thus, we investigated if low glycogen storage would be related to high glycogenolysis via PEPCK activation by analyzing the *Pepck* transcript. We found an increase in its expression at ZT8 (Figure 2F), at the same time point when glycogen storage was reduced in the liver of mice lacking the TRPV1 channel. Given that hepatic glycogen storage was reduced and *Pepck* transcript was increased at ZT8 in the absence of TRPV1 channels, we next evaluated which protein could be altered in the liver of *Trpv1* KO mice by analyzing the proteome of both genotypes.

Overall, we demonstrated that the lack of TRPV1 channels leads to the decreased expression of clock genes and the increased expression of metabolic genes at ZT8 compared to WT mice. The time-dependent functions in the liver have been often observed since some components of glucose hepatic production are increased at the light phase [18,20]. For instance, hepatic glucose, glucose-6-phosphate, and oligosaccharides of glycogen breakdown (represented by maltopentaose levels) accumulate during the light phase [20]. These time-specific changes in glucose metabolism are coincident with high glycogenolysis and gluconeogenesis in the liver during fasting. Thus, our data indicate that reduced expression of *Per1* and *Bmal1* at ZT 8, observed in the absence of TRPV1 channels, could directly influence these two processes. In addition, the reduced glycogen storage at ZT8 in the liver of *Trpv1* KO mice reinforces the high hepatic glucose production in the light phase.

Notably, it was reported that *Bmal1* and *Glut2* have an antiphase relationship, in which at ZT8, *Bmal*1 expression is down, while *Glut2* displays a peak expression at ZT12 [20]. Interestingly, *Glut2* has an antiphase relationship with hepatic glucose production since at the end of the light phase (ZT8), when hepatic glucose is high, the expression of *Glut2* is increased to enable the export of hepatic glucose to the circulation. Thus, *Glut2* reaches its peak expression at ZT12, the moment when the eating phase starts, and then hepatic glucose goes down [20]. Therefore, we highlight that the gene expression changes related to energy metabolism, seen in the absence of TRPV1 channels, may be associated with reduced *Bmal1* expression at ZT8 and/or directly triggered by the lack of the channel in the regulation of metabolic processes. Considering that such changes were not observed in the gene expression of *Bmal1*, *Glut2*, *Pepck*, and *G6pase* at ZT20 in the liver of *Trpv1* KO mice compared to WT mice, we hypothesized that the TRPV1 channel plays an important role under physiological fasting conditions.

### 2.2. Lack of TRPV1 Channel Affects Proteome and Disrupts Biological Functionality in Liver

**Figure 3:**
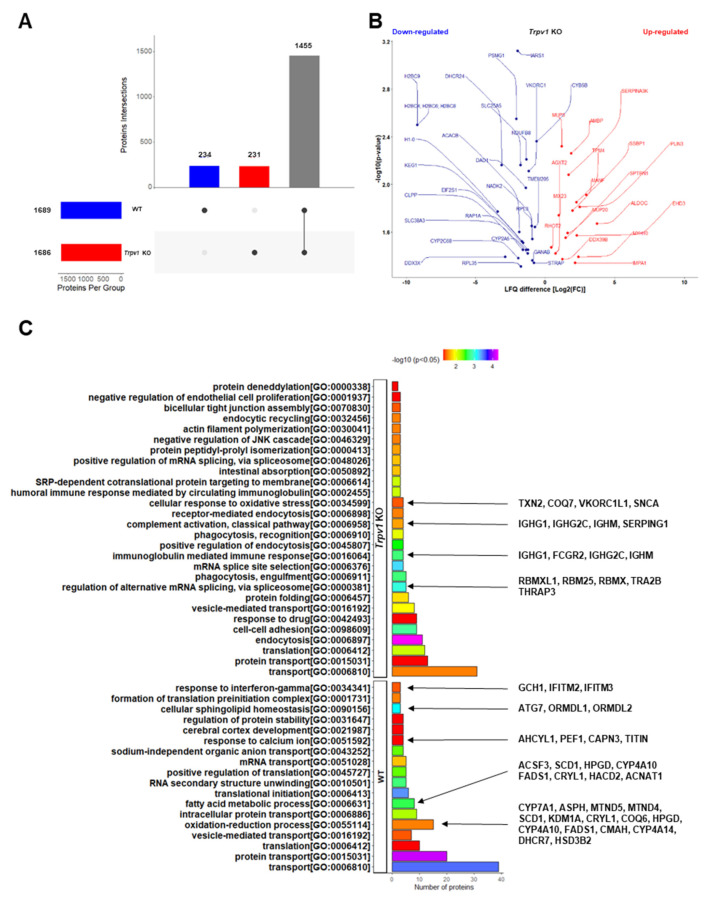
The lack of a TRPV1 channel affects hepatic functionality, as revealed by the integration data of liver proteomes. (**A**) The UpSet plot presents the overlapping of proteins found in the liver proteome of *Trpv1* KO and WT mice. (**B**) Scatter plots show the distribution of proteins shared between liver proteomes, indicating downregulated and upregulated proteins in *Trpv1* KO mice. LFQ difference denotes log2 (fold change) among groups. (**C**) Gene ontology biological process (GO-BP) enrichment analyses of differentially identified proteins in the liver proteomes using the annotation of the DAVID database (*p* < 0.05) are highlighted for lipid metabolism, RNA processing, oxidative stress, and inflammation functional processes that were exclusively expressed in WT or *Trpv1* KO animals.

To explore the mechanisms underlying the fasting metabolic adaptation in the absence of TRPV1 channels, we used a quantitative relative-based mass spectrometry approach (label-free quantification, LFQ) to investigate changes in the liver proteomic signature of *Trpv1* KO mice compared to WT animals at ZT8 (Supplementary Information File S1, Tables S1–S3). We found 1920 proteins in both proteomes, of which 234 and 231 proteins were unique to the liver of WT and *Trpv1* KO mice, respectively (Figure 3A). We found that 1455 proteins were shared between the proteomes, with 26 proteins downregulated and 17 upregulated in the liver of *Trpv1* KO mice in comparison to WT mice (Figure 3B).

Differential proteins encoded in the liver of WT and *Trpv1* KO mice could indicate functional differences induced by the absence of TRPV1 channels. In this regard, we highlighted the downregulation of core components of nucleosomes (H2BC9, H2BC4, and H1-0) and lipid metabolism (DHCR24 and DDX3X) in the absence of TRPV1 channels. It has been shown that nucleosomes serve as a regulator of transcription since their interaction with gene promoters prevents the initiation of transcription [41]. Considering that in the absence of TRPV1 channels, reduced nucleosome proteins were found, we speculated whether transcription regulation could have been affected. In fact, we found increased gene expression of the majority of the hepatic metabolic genes analyzed here (Figure 1 and Figure 2).

In addition, DHCR24, a protein involved in an anti-inflammatory and pro-resolving phenotype (accelerating inflammation resolution) [42], was reduced in the absence of TRPV1 channels (Figure 3B), which could be related to a more inflammatory phenotype. RNA helicase DDX3X is involved in a variety of transcript metabolic regulations [43], including lipid metabolism [44]. Downregulation of DDX3X leads to decreased protein and transcript expression of microsomal triglyceride transfer protein (MTP) and, therefore, may enable the blocking of lipoprotein assembly and the inhibition of lipid export, which is a leading cause of lipid accumulation [44]. We found a reduction of this protein in the absence of TRPV1 channels (Figure 3B). In this sense, we found high expression of *Pparα* and *Pparγ* (Figure 1B,C) in the liver of *Trpv1* KO mice, two important transcription factors involved in lipid metabolism [26].

Some of the most significantly increased proteins (ALDOC, SSBP1, PLIN3, EHD3) (Figure 3B) participate in the same molecular mechanisms as some decreased proteins in processes that converge to the establishment of the liver phenotype in *Trpv1* KO mice. For instance, some increased proteins, in addition to decreased DHCR24 and DDX3X, also suggest a phenotype related to fatty acid metabolism changes in the absence of TRPV1 channels. Among them, ALDOC, an aldolase family member that has a central role not just in glycolysis but also in gluconeogenesis pathways, was upregulated 3.4-fold (log2), indicating glucose and glycerolipid metabolism changes in the liver of *Trpv1* KO mice [45,46]. PLIN3 is involved in lipid storage, mobilization, and droplet biogenesis, and it has also been associated with hepatic steatosis [47]. This protein also regulates early-endosome-to-Golgi transport [48], similar to EHD3 [49]. In parallel, SSBP1, which was also increased in *Trpv1* KO mice (Figure 3B), is involved in the replication and maintenance of mitochondrial DNA, DNA repair, and cell protection from proteotoxic stresses [50].

To assess in depth the hepatic functional dysregulation caused by the lack of TRPV1 channels, we next conducted a gene ontology biological process (GO-BP) enrichment analysis using exclusively identified proteins of each group. We found that 43 differential GO-BPs were evidenced in both groups (Figure 3C). The differential GO-BP of “regulation of alternative mRNA splicing, via spliceosome” in the liver of *Trpv1* KO mice indicated enhanced RNA processing, which is in line with the downregulation of H2BC9, H2BC4, and H1-0 (core components of nucleosomes, Figure 3B). Proteins clustered in this enriched GO-BP may regulate a variety of post-transcriptional processes (e.g., alternative splicing, nucleocytoplasmic transport, translation, and degradation) under stress conditions and cellular reprogramming [51,52]. Of these, THRAP3 promotes the binding of CLOCK-BMAL1 to DNA and links it to the basic transcriptional machinery [53]; it is also required for the DNA damage response, control of mRNA splicing, and nuclear mRNA degradation [54]. In addition, we evidenced “cellular response to oxidative stress”, which might be intimately linked to DNA damage and inflammation, as demonstrated by the participation of TXN2 in previous studies [55,56]. In this context, the immune response mediated by immunoglobulin in *Trpv1* KO mice seems to indicate inflammatory status [57,58], which is in line with reduced DHCR24 involved in the anti-inflammatory phenotype (Figure 3B).

In contrast to enriched immune responses mediated by immunoglobulin in the liver of *Trpv1* KO mice, we observed that “response to interferon-gamma” was enriched only in the WT liver. It has been demonstrated that interferon-gamma deficiency attenuates hepatic inflammation in a steatohepatitis model [59]. Thus, the lack of proteins related to “response to interferon-gamma”, associated with the compensatory hepatoprotective mechanism shown in the liver of *Trpv1* KO mice, may be suggestive of a protective effect displayed against inflammation mediated by the absence of TRPV1 channels. In this line, it was demonstrated that decreased HPGD expression in hepatic endothelial cells is related to hepatoprotective effects in response to liver injury [60]. Indeed, we identified the HPGD protein in the liver of WT mice but not in *Trpv1* KO mice (Figure 3B), reinforcing our hypothesis that *Trpv1* KO mice display an inflammatory status and subsequent hepatoprotective response. Other proteins that participate in the fatty acid metabolism identified in the liver of WT mice (ACNAT1, FADS1, and NADK2) have been described to prevent inflammation and oxidative stress [61,62,63] but were not found in the *Trpv1* KO liver (Figure 3C).

Some of the enriched GO-BPs in the liver of WT mice are related to metabolism responses, including cellular sphingolipid homeostasis, the fatty acid metabolic process, and the oxidation-reduction process (Figure 3C), indicating, mainly, metabolic reprogramming of the lipid metabolism in the absence of TRPV1 channels. Among the associated proteins, mitochondrial enzyme malonate-CoA ligase (ACSF3) induces malonyl-CoA production from malonate, a toxic metabolic product from mitochondrial metabolism [64]. Thus, the fact that ACSF3 was not identified in the liver of *Trpv1* KO mice could be related to a possible reduction in malonyl-CoA biosynthesis. Importantly, the acetyl-CoA carboxylase 2 (ACACB, also known as ACC2), a mitochondrial enzyme that catalyzes the carboxylation of acetyl-CoA to form malonyl-CoA [65], was reduced 1.85-fold (log2) in *Trpv1* KO liver (Figure 3B). In line with these findings, reduced malonyl-CoA availability would lead to increased CPT1 activity, followed by increased fatty acid oxidation (FAO) and reduced lipogenesis [66]. Next, we identified three fatty-acid-binding proteins (FABP1, FABP4, and FABP5) in the WT proteome and four proteoforms (FABP1, FABP2, FABP4, and FABP5) in the *Trpv1* KO proteome. These proteoforms bind long-chain FA with high affinity and are involved in lipid uptake and FA transport [67]. Among these, FABP2 is associated with lipid droplet accumulation in HepG2 cells, being a downstream target of PPARγ as well [68]. It is also related to lipid metabolism changes in the liver of type 2 diabetes mellitus in a nonalcoholic fatty mice model [69].

Hepatic lipid accumulation occurs when the uptake of circulating FA and de novo lipogenesis surpasses the FAO and export of lipids in very-low-density lipoproteins (VLDLs) [70]. Based on our findings in the liver proteomics analysis of *Trpv1* KO mice compared with WT mice, as demonstrated by DDX3X (less lipid export), ACACB and ACSF3 (less malonyl-CoA and subsequently higher FAO), FABP2 (high FA uptake) and PLIN3 (lipid availability), we suggest that the liver of *Trpv1* KO mice could have greater FA availability to beta-oxidation to support a boost in gluconeogenesis, thus preventing lipid accumulation. In fact, hepatic triglyceride levels in WT did not differ from *Trpv1* KO mice [35].

Interestingly, proteins involved in glucose metabolism in *Trpv1* KO and WT mice function in opposite directions. UDP galactose-4-epimerase (GALE), an enzyme that participates in the synthesis of glycoproteins and glycolipids, has been found in the liver of *Trpv1* KO mice. The hepatic overexpression of GALE has been reported to enhance gluconeogenesis from pyruvate [71]. On the other hand, glucosamine-6-phosphate isomerase 1 (GNPDA1) has been found only in the liver of WT mice. This enzyme provides a source of energy in the form of phosphosugars derived from the catabolism of glycoproteins and glycolipids and transfers them into the glycolytic pathway [72].

Taken together, the above data seem to indicate that the absence of TRPV1 channels alters gene expression and RNA processing and induces inflammatory features, oxidative stress, and an increase in FAO to support more substrates for gluconeogenesis, resulting in changes in glucose metabolism.

### 2.3. Protein–Protein Interaction Network Correlates the TRPV1 Channel and Hepatic Glucose Metabolism

**Figure 4:**
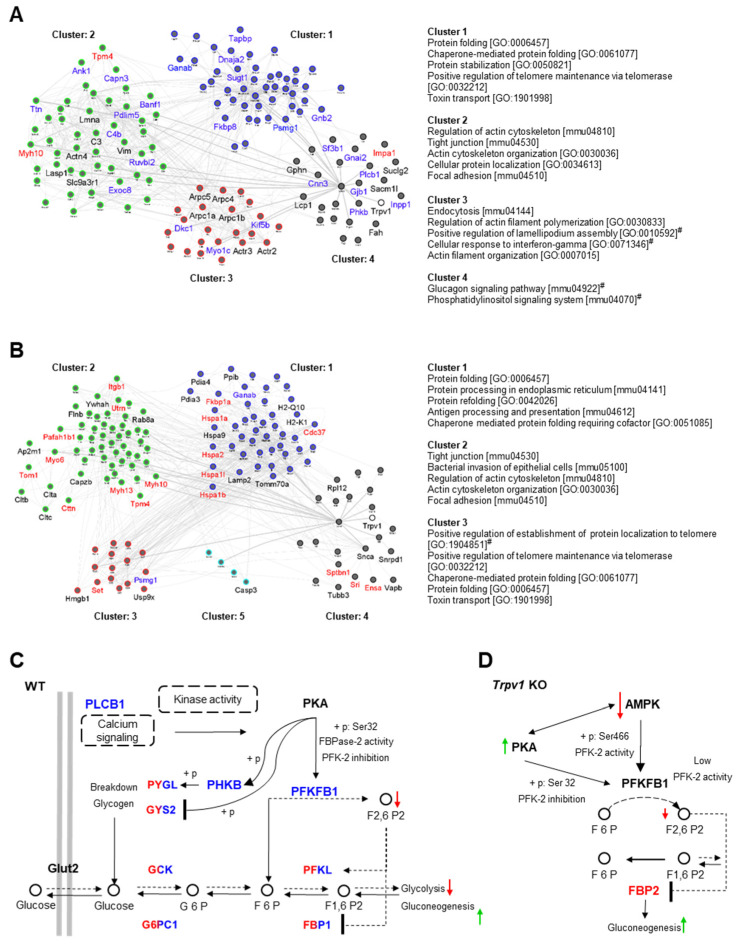
Network propagation highlights important liver clustering pathways and biological networks in the absence of TRPV1 channels. Network propagation of the TRPV1 channel in the liver of WT (**A**) and *Trpv1* KO mice (**B**). TRPV1 protein input under each network served as a driver for network propagation in *Trpv1* KO and WT. Separately propagated subnetworks with 141 proteins found to be significant in the propagation were clustered into smaller subnetworks and annotated using integrated enrichment analysis (GO-BP and KEGG pathway) to identify functional networks that are different between two propagated subnetworks (EnrichmentMap FDR < 0.01). Node names represent the proteins exclusively found in each propagated subnetwork; black for proteins shared between *Trpv1* KO and WT proteomes; blue for proteins only found or upregulated in the WT proteome; red for proteins only found or upregulated in the *Trpv1* KO proteome. Cluster title indicates the top five most significant biological function(s) for each cluster, and (#) refers to the KEGG pathway/GO-BP annotations exclusively enriched in each propagated subnetwork. (**C**) Framework of propagated network integration for enriched glucagon signaling pathway from KEGG in the WT proteome. (**D**) Framework shows proposed modulation of FBP-2 by PKA and AMPK kinases in *Trpv1* KO mice resulting in more active gluconeogenesis than in WT mice. Blue nodes for proteins only found in the WT proteome, dual colors (blue/red) for proteins shared among proteomes, and black for proteins not found in the proteomic data. (+p) refers to phosphorylation and (−p) to dephosphorylation.

We constructed a protein–protein interaction network (PPIN) based on a physical interaction complex of all proteins identified in each proteome and by aggregating them to the TRPV1 protein, although this key protein was not found in our analysis. Further, we removed nodes (proteins) disconnected from the rest of the network. The PPINs assembled through WT and *Trpv1* KO proteomes, with 1389 proteins/16,394 interactions and 1415 proteins/16,633 interactions, respectively, were used to build a TRPV1-driven subnetwork based on the oriented network method (diffusion) to explore connectivity patterns directly linked to it. The resulting oriented subnetworks had 141 proteins (~10%) from PPINs, of which 100 were overlapped among them. Each cluster of protein interaction was submitted to enrichment analysis using the GO biological process and KEGG pathways, where most of the enriched GO terms and signaling pathways share subnetworks (Figure 4A).

Using this approach, we identified that glucagon signaling was enriched in cluster 4 of the PPIN from the liver of WT mice (Figure 4A). This was an important and unexpected finding since glucagon signaling in *Trpv1* KO mice was not identified (Figure 4B) in a similar way as described for WT mice. Cluster 4 from the WT network included “glucagon signaling pathway” (PYGL, PYGM, CALM3, PHKB, PLCB1) and “phosphatidylinositol signaling system” (CALM3, PLCB1, SACM1L, INPP1, IMPA1). Calmodulin-3 (CALM3) is a key protein in both clusters and is connected to TRPV1; however, connections with proteins exclusively identified in the WT proteome (PLCB1 and PHKB) enabled glucagon pathway enrichment. In a downstream pathway, PLCB1 interacts with INPP1, SACM1L, and IMPA1 in phosphatidylinositol signaling. Thus, the relationship shown here proposes that TRPV1 absence leads to PLCB1 signaling impairment and, thus, directly influences associated pathways such as glucose metabolism. Indeed, the relationship between TRPV1 and PLCB1 has been reported in several studies [73,74,75,76].

Given that the glucagon signaling pathway (KEGG ID: mmu04922) is directly related to the TRPV1 channel, we focused on further analysis of this pathway to maximize insight into the effect of TRPV1 absence on glycogenolysis/gluconeogenesis signaling pathways. We found that of the 25 genes mapped for this database, 21 proteins from WT and *Trpv1* KO were overlapping. The proteins phospholipase C beta 1 (PLCB1), phosphorylase kinase subunit beta (PHKB), and 6-phosphofructo-2-kinase/fructose-2,6-bisphosphatase 1 (PFKFB1, known as PFK/FBPase-1) were exclusively identified in the WT proteome, while fructose-1,6-bisphosphatase isozyme 2 (FBP2) was uniquely identified in the *Trpv1* KO proteome.

Based on the direct visualization of this pathway and the resulting outputs to glucagon stimulus reported by the KEGG database, we reassembled this signaling pathway using shared proteins among groups and exclusively identified proteins in the liver of WT mice for a better understanding of glycogenolysis and gluconeogenesis in the absence of TRPV1 channels. It was observed that cyclic AMP-dependent protein kinase (PKA) displays a key role in hepatic glucose regulation, phosphorylating PFKFB1 and PHKB proteins (Figure 4C). In response to glucagon, PKA phosphorylates Ser32 of PFKFB1 in the liver, which, in turn, leads to PFK-2 inactivation and FBPase-2 activation to reduce D-fructose 2,6-bisphosphate (F2,6-P2), thus increasing gluconeogenesis [77]. As F2,6-P2 is a powerful allosteric activator of 6-phosphofructo-1-kinase (PFK-1) to stimulate glycolysis and an inhibitor of fructose-1,6-bisphosphatase (FBP), a regulatory enzyme of gluconeogenesis, glucagon decreases the concentration of hepatic F2,6-P2, allowing gluconeogenesis activation [78].

On the other hand, we also investigated the role of the FBP-2 protein and upstream kinases that indirectly regulate its activity in the signaling pathway of *Trpv1* KO liver. In this signaling pathway, PKA and AMP-activated protein kinase (AMPK) represent the main regulatory proteins of PFKFB1 and, subsequently, FBP-2 (Figure 4D). Unlike the PKA-mediated mechanism in the liver, activation of AMPK leads to the phosphorylation and activation of PFK-2 (heart isoform), increasing F2,6-P2 and stimulating glycolysis [79]. In addition, AMPK activation has antagonistic mechanisms to PKA activity in the liver. An increase in the intracellular AMP level not only increases AMPK activity but also inhibits adenylyl cyclase, reducing the ability of PKA to promote gluconeogenesis [80]. Interestingly, liver AMPK activity is impaired upon glucose starvation of *Trpv1* KO mice [12], which suggests decreased F2,6-P2 levels in this genotype. In addition, the relationship between decreased AMPK activity and the downregulation of ACC2 in the liver of the *Trpv1* KO mice shown here can be found in the glucagon signaling pathway from the KEGG database.

Since FBP-2 protein, a regulatory enzyme of gluconeogenesis that converts F1,6-P2 to F6P for hepatic glucose production, was positively regulated in the liver of *Trpv1* KO mice, it is suggestive that a high gluconeogenesis rate is found in the absence of TRPV1 channels. Increased gluconeogenesis in *Trpv1* KO mice might also be supported by the fact that the lack of TRPV1 channels would lead to deregulated Ca^2+^ signaling. Ca^2+^ negatively regulates FBPase [81], and it may inhibit adenylyl cyclases 5 and 6 [82]. In this sense, TRPV1 channel absence and subsequently disrupted Ca^2+^ signaling could contribute to higher FBPase activation through increased PKA-dependent phosphorylation. In fact, constitutive activation of PKA in the liver results in fasting hyperglycemia and evidence of increased glycogenolysis and gluconeogenesis [83].

### 2.4. Differential Interactions of Kinases and Phosphatases Underline Changes in Hepatic Glycogen Metabolism in Trpv1 KO Mice

**Figure 5:**
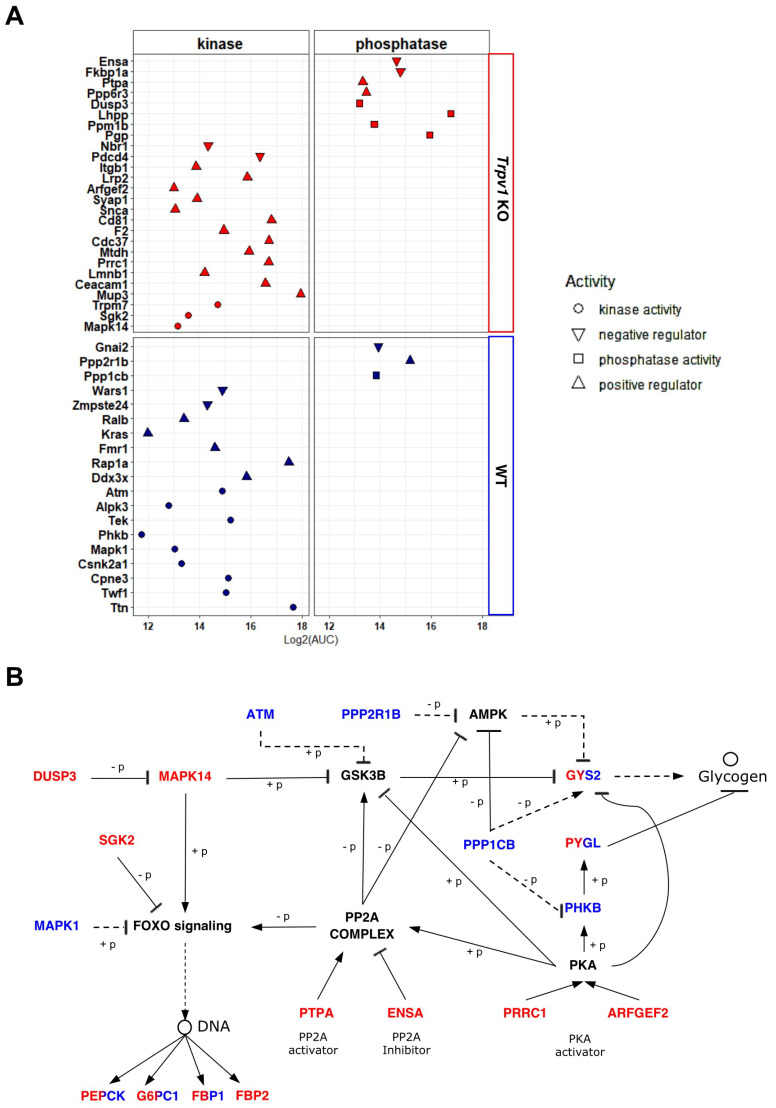
Network integration between kinases and phosphatases for modulation of hepatic glycogenesis/glycogenolysis in the absence of TRPV1 channels. (**A**) Mapping of kinases, phosphatases, and their regulatory proteins exclusively identified in the liver of *Trpv1* KO or WT mice or those that showed significant differences in the relative quantification by the LFQ approach. (**B**) Differentially identified kinases, phosphatases, and regulatory proteins of each proteome were integrated into the same network to identify mechanisms that were different between *Trpv1* KO and WT livers. Blue color for proteins only found in the WT proteome, dual colors (blue/red) for proteins shared among proteomes, and black for proteins not found in the proteomic data. (+p) refers to phosphorylation and (−p) to dephosphorylation.

We investigated the participation of exclusively identified kinases and phosphatases in each proteome in the modulation of glycogenolysis/glycogenesis. Using gene ontology annotation from the Uniprot database, we mapped MAPK14, SGK2, and TRPM7 kinases and PGP, PPM1B, LHPP, and DUSP3 phosphatases in *Trpv1* KO mice, while in the liver of WT mice, TTN, TWF1, CPNE3, CSNK2A1, MAPK1, TEK, ALPK3, PHKB, and ATM kinases and phosphatase PPP1CB were identified. We also examined proteins that improve or impair the kinase/phosphatase activity (Figure 5A). After that, we used the PhosphoSitePlus database (https://www.phosphosite.org/, accessed on 19 June 2022) with a setting to mouse, KEGG pathway annotation, and literature data to map the relationship between kinases/phosphatases and their upstream regulatory proteins and downstream targets and glycogenolysis/glycogenesis. Using these strategies, 11 proteins related to glycogenolysis/glycogenesis pathways were mapped (Table 2).

For a better understanding of the functionality of these mapped proteins in the glycogenolysis pathways, we assembly a PPIN, remaking connections among the mapped proteins that break down glycogen and are expected to have high activity at ZT8. To avoid a gap of connection in the PPIN, we then inserted upstream proteins of hepatic glycogen (i.e., GYS2 and PYGL) that were identified in both proteomes and proteins not found in the proteome data (PKA, AMPK, GSK3B, and FOXO) that are associated with downstream signaling of the mapped proteins (Figure 5B). Interaction of some kinases with upstream phosphatases was observed, which seems to be required to attenuate kinase activity by negative feedback (e.g., DUSP3 andMAPK14 in *Trpv1* KO mice and PPP1CB and PHKB in WT mice). Moreover, our results show that the dephosphorylation and phosphorylation of target proteins are a response to the increased activity of some positive regulatory proteins and, therefore, trigger an important role in downstream signaling.

Taken together, the results obtained with differentially identified kinases, phosphatases, and regulatory proteins in *Trpv1* KO and WT mice indicate that changes observed in the *Trpv1* KO mice orchestrate an increased breakdown of glycogen. In this context, PKA activity is positively regulated by PRRC1 [84] and ARFGEF2 (also known as BIG2), which acts as an A kinase-anchoring protein (AKAP) [85]. Then, PKA activates protein phosphatase 2A (PP2A complex) [86]. The PP2A phosphatase activator (PTPA) also stimulates PP2A activity in the presence of Mg^2+^ and Zn^2+^ [87]. Transient receptor potential cation channel subfamily M member 7 (TRPM7) promotes Zn^2+^ release from intracellular stores to regulate ROS signaling in oxidative stress responses [88], as indicated by functional enrichment analysis in *Trpv1* KO mice. Intriguingly, ENSA, a PP2A inhibitor [89], was also found in the liver of *Trpv1* KO mice (Figure 5A), and it seems to act as an “on–off” switch of the phosphatase complex. Once PP2A is active, it mediates the dephosphorylation and inhibition of AMPK and the dephosphorylation and activation of the FoxO1 transcription factor [90]. Among FoxO signaling targeted genes, we observed an increase in the expression of *G6pc1* and *Pck1* (also known as *Pepck*) at the transcription level in the absence of TRPV1 channels (Figure 2), which nevertheless did not show significant differences in our proteomic data.

Furthermore, PP2A has also been described as a GSK3B activator [91]. However, it is important to mention that multiple players may regulate GSK3B, as shown in Figure 5B. Thus, the role of the TRPV1 channel in GSK3B phosphorylation is yet unclear due to the many upstream kinases and phosphatases involved in this signaling.

Contrary to *Trpv1* KO mice, PPP1CB phosphatase and PHKB kinase were identified in the liver of WT mice, in which PPP1CB acts upstream of PHKB to inhibit it and subsequently promotes the inhibition of PYGL (Figure 5B). PPP1CB is required to regulate glycogen synthesis because it not only inhibits PYGL but also activates GYS2 [92]. We speculate that the PPP1CB may participate in the attenuation of breakdown glycogen magnitude and enable glucose homeostasis at ZT8.

Zhong and collaborators [8] demonstrated that the blood glucose of *Trpv1* KO mice has no significant differences in basal conditions when compared to WT mice; however, there was a significantly higher blood glucose rate in *Trpv1* KO mice after glucose stimulus. We believe that the initial difference in blood glucose level could not be observed because the daily switch of glycogen metabolism is evidenced by elevated glycogen breakdown, with peaks of glycolytic/gluconeogenic metabolites at ZT8 [18]. Although multiple layers of regulation are required for glycogen metabolism, our results strongly suggest that the lack of TRPV1 channels promotes the increase of glycogenolysis, gluconeogenesis, and FOXO signaling through the PP2A–PKA axis and, thereby, enhances hepatic glucose production.

## 3. Material and Methods

### 3.1. In Vivo Procedures

In vivo experimental procedures were conducted on a total of 20 male mice ranging from 3 to 4 months old: 10 C57BL/6J wild-type (WT) and 10 *Trpv1* KO in C57BL/6J background mice provided by the Department of Physiology, University of Sao Paulo (USP), originally obtained from Jackson Laboratory (B6.129X1-Trpv1*^tm1Jul/^*J, Stock No.: 003770). The lack of *Trpv1* channels was confirmed by quantitative PCR (data not shown). Experimental procedures were performed according to the protocol approved by the Ethics Committee for Animal Use (CEUA IB/USP, number 255/16). All methods were carried out in accordance with relevant guidelines and regulations.

Mice were singularly housed in standard propylene cages with access to food and water ad libitum, kept at 22 °C ± 1 under a 12:12 light/dark cycle (800–1000 lux white LED light, ranging from 420 to 750 nm, lights on at 7 AM (ZT 0) and off at 7 PM (ZT 12)). After a 7-day acclimation, the animals were kept in the same conditions for additional 14 days. On the 15th day, the mice were euthanized with CO_2_ at ZTs 8 and 20, and death was assured by cervical dislocation. At ZT 20, all experimental procedures were carried out with the assistance of night goggles in complete darkness. The liver from 5 WT and 5 *Trpv1* KO mice at each time point (ZT8 and ZT20) was removed for RNA extraction, quantitative PCR (qPCR), and glycogen quantification. Proteomics analyses were performed using 4 biological replicates of each group at ZT8, as described below. The time points of euthanasia were chosen based on the fact that peaks of glycolytic/gluconeogenic metabolites and maximal BMAL1-DNA binding occur at ZT8 [18]. To compare time-dependent changes between light and dark phases, we also analyzed gene expression and glycogen storage 12 h later, which corresponds to ZT20.

### 3.2. Total RNA Extraction and Reverse Transcriptase–Polymerase Chain Reaction (RT–PCR)

Small liver fragments were homogenized in TRIzol (Thermo Fisher Scientific, Waltham, MA, USA), and total RNA was extracted and purified according to the manufacturer’s instructions. Total RNA concentration (OD_260_) was determined using a spectrophotometer (Nanodrop, Wilmington, DE, USA), and 1 μg was subject to reverse transcription with Superscript III Reverse Transcriptase, random hexamer primers, and other reagents according to the manufacturer’s instructions (Thermo Fisher Scientific, Waltham, MA, USA), generating complementary DNA (cDNA).

### 3.3. Quantitative PCR (qPCR)

cDNA was subject to quantitative PCR reactions, as previously described [13,14], using a species-specific pair of primers and a probe (Table 3) based on sequences obtained from GenBank (http://www.ncbi.nlm.nih.gov/genbank, accessed on 15 November 2019). Primers and probes were designed as spanning introns by the Primer Express program (Life Technologies, Carlsbad, CA, USA), and synthesized by IDT (Coralville, IA, USA); 18S ribosomal RNA was used to normalize the values of the genes of interest. Prior to this selection, we ascertained that 18S rRNA did not vary among time points (data not shown).

For qPCR using the *Taqman* multiplex protocol for simultaneous analysis of *Per1, Bmal1*, and 18S rRNA, the solutions contained primers (300 nM for the genes of interest and 50 nM for 18S rRNA) and probes (200 nM for the genes of interest and 50 nM for 18S rRNA) (Table 3), PowerMix 2X (Bio-Rad Laboratories, Hercules, CA, USA), and cDNA in triplicate for each sample. Individual assays for *Trpv1*, *Akt*, *G6pase*, *Glut2*, *Gsk3β*, *Pparα*, *Pparγ, Pepck,* or 18S rRNA were prepared with primers at 300 nM (Table 3), KAPA SYBR FAST 2x (Kapa Biosystems, Wilmington, MA, USA), and cDNA in duplicates for each sample. Non-template wells were routinely included.

Reactions were carried out in an i5 thermocycler (Bio-Rad Laboratories, Hercules, CA, USA) in the following conditions: for multiplex assays, 3 min at 95 °C, followed by 45 cycles of 15 s at 95 °C and 60 s at 60 °C; for SYBR Green assays, 10 min at 95 °C, followed by 45 cycles of 15 s at 95 °C, 1 min at 60 °C, and 80 cycles of 10 s, starting at 55 °C with a gradual increase of 0.5 °C.

### 3.4. Gene Expression Data Analysis

Gene expression assays were quantified according to the 2^−ΔΔCT^ method [93]. All values were normalized by 18S ribosomal RNA, and the resulting minimal mean value obtained from WT mice was subtracted from all other values for both WT and *Trpv1* KO mice, obtaining ΔΔC_T_. Then, ΔΔC_T_ was used as a negative exponential of base 2 (2^−ΔΔCT^), providing the relative expression of the gene between genotypes and between time points within each genotype.

The log values were obtained from 5 animals, and the median, quantiles, and maximum and minimum expression values of gene transcripts normalized by 18S ribosomal RNA and expressed relative to the minimum mean value of WT mice. were plotted in graphs. Student’s *t*-test was used to compare *Trpv1* gene expression between ZT 8 and ZT 20 in WT mice. To determine the differences between time points of the same genotype or among genotypes at the same time point, logarithmic data were compared by two-way ANOVA, followed by the Bonferroni post-test; *p* < 0.05 was established to reject the null hypothesis. All statistical analyses were carried out in Prism 7.0 (GraphPad 7.0, San Diego, CA, USA).

### 3.5. Glycogen Quantification

To measure the amount of liver glycogen, 250 mg of liver was processed as described elsewhere [94]. The absorbance was measured at a wavelength of 650 nm in duplicate for each sample in a plate reader (Biotek Sinergy H1, Winooski, VT, USA). Hepatic glycogen quantification was calculated using the absorbance of the samples in relation to tissue mass (milligrams) and normalized by the glucose concentration curve.

### 3.6. Sample Preparation for Mass Spectrometry (MS) Analysis

Frozen livers of *Trpv1* KO and WT mice (*n* = 4 per group) euthanized at ZT8 were mechanically homogenized in 100 mM Tris-HCl, pH 7.8, 3% SDS, and 150 mM NaCl supplemented with protease and phosphatase inhibitors (Roche, Indianapolis, IN, USA) using a bead blaster refrigerated homogenizer (Benchmark Scientific, Sayreville, NJ, USA) at a maximum speed of 3650 rpm with three cycles of 30 s on-and-off at 4 °C. Samples were then sonicated for three cycles (20 s bursts with 20 s pauses) and centrifuged at 16,000× *g* for 10 min at 4 °C. The supernatants were collected, and the protein concentration was measured using the Pierce BCA protein assay in technical duplicates for each sample (Thermo Scientific, Waltham, MA, USA). Aliquots of SDS-lysates containing 300 µg of total protein from each sample were processed according to the filter-aided sample preparation (FASP) method using Microcon 10 kDa centrifugal filter units (Merck Millipore, Burlington, MA, USA) at 10,000× *g* for 50 min at 20 °C [95,96,97]. To remove SDS, filters containing proteins were washed with urea buffer. Next, Trypsin/LysC Mix (Promega, Madison, WI, USA) was added to the filters at an enzyme-to-protein ratio of 1:100 (*w*/*w*) and incubated for 16 h at 37 °C in a thermo-mixer at 600 rpm. Following protein digestion, peptides were filtered through the membrane using 3% acetonitrile in 0.1% trifluoroacetic acid (TFA) and purified with reversed-phase chromatography using C18 micro-pipette tips (TopTip^TM^, PolyLC, Columbia, MD, USA) according to the manufacturer’s instructions. Peptides were dried in a vacuum concentrator and stored at −20 °C.

### 3.7. LC–ESI–MS/MS Analysis

Dried peptides were recovered in 20 µL of aqueous 0.1% formic acid and analyzed by online nanoflow LC–ESI–MS/MS using an ACQUITY UPLC system (Waters, Milford, MA, USA) coupled online to a maXis 3G quadrupole time-of-flight (Q-TOF) mass spectrometer (Bruker Daltonics, Bremen, Germany), equipped with a Captive Spray nanoelectrospray source. One µL of the sample was injected, and the peptide mixture was loaded onto a trap column (nanoAcquity UPLC^®^ 2G-V/M Trap 5 µm Symmetry^®^ C18, 180 µm × 20 mm, Waters, Milford, MA, USA) for 3 min at a flow rate of 7 µL/min of aqueous 0.1% formic acid. Peptides were separated on an analytical column (nanoAcquity UPLC^®^ 1.8 µm HSS T3, 75 µm × 200 mm, Waters, Milford, MA, USA) using a 240 min gradient from 2% to 85% of 0.1% formic acid in acetonitrile (1 min at 2%, 209 min 2–30%, 10 min 30–85%, 5 min wash at 85%, 5 min 85–2%, 10 min equilibration at 2%). The flow rate was set at 200 nL/min. Eluted peptides were analyzed in a maXis 3G (Q-TOF) mass spectrometer operated in the positive mode under a data-dependent acquisition manner in the m/z range of 150–2200. Precursor ions were fragmented with collision-induced dissociation (CID).

### 3.8. MS Data Analysis and Statistical Analysis

The raw files (.d) were loaded into PEAKS Studio 8.5 software (Bioinformatics Solution Inc., Waterloo, ON, Canada), and the PEAKS standard workflow (de novo peptide sequencing, PeaksDB, PeaksPTM, and SPIDER) was applied to the identification of proteins [98,99]. MS/MS spectra were searched against the *Mus musculus* reference proteome database available at UniprotKB (Proteome ID UP000000589—21,986 protein entries, download one protein sequence per gene, release date Dec 2021). The following parameters were used: precursor mass tolerance of 25 ppm; fragment mass tolerance at 0.02 Da; trypsin was set as the specific enzyme, and up to two missed cleavages were required; carbamidomethylation on cysteine (+57.02 Da) as a fixed modification, oxidation on methionine (+15.99 Da), and acetylation on protein N-terminus (+42.01 Da) were set as variable modifications, with maximal 3 modifications per peptide in SPIDER outcomes. Significance score of −10lgP > 20 (*p*-value <0.01) for proteins and peptides was applied, and at least 1 unique peptide was required for protein identifications. Proteins were considered to be validated in an experimental group when they were identified in at least two out of the four biological replicates of a given group. The PEAKS Q module was applied (label-free quantification method) to the database search outcome, and the normalization factors based on total-ion count (TIC) were obtained. The normalization factor obtained for each sample was employed to calculate the normalized areas of proteins. Normalized protein areas have been log2-transformed, and the values were subject to Student’s *t*-test, in which *p*-values < 0.05, and fold changes ≥1.2 (log2 (FC) ≤ −0.26 or ≥0.26) were selected as cut-off points of significance to the differential relative abundance of proteins.

### 3.9. MS Data Integration, Network Propagation, and Functional Analysis

To identify biological processes represented by the differentially identified proteins in each proteome, we carried out a functional enrichment analysis of gene ontology biological processes (GO-BPs) using the Database for Annotation, Visualization, and Integrated Discovery (DAVID v.6.8) functional annotation tool [100].

All proteins identified in each proteome were considered for the generation of protein–protein interaction networks (PPINs) of WT and *Trpv1* KO mice. UniProt IDs from the protein list were used to perform a multiple protein search in STRING DB v.11.5 (https://string-db.org/, accessed on 19 June 2022) [101]. The chosen protein to set this analysis was based on those that were indicated as part of a physical interaction complex and those that presented a minimum required interaction score of 0.4 (medium confidence score). The resulting interaction network was imported into Cytoscape v.3.8.2 (Boston, MA, USA) [102] for visualization. Then, we conducted a supervised network propagation considering protein–protein interactions and hierarchical relationships using Cytoscape plug-in diffusion [103]. Network propagation is an important method to identify and highlight biological signals within a dataset since it can amplify the association between proteins that lie in network proximity and enable a reduction in the number of paths in the network to guide subsequent analysis focused on targets [104,105]. As the goal of this analysis is to find a subnetwork that is related to the TRPV1 channel and may affect glycogen metabolism, we chose the TRPV1 protein for driving the underlying network to it, using a cut-off point of the top 10% of the most relevant proteins.

Clusters in the subnetwork were identified using the GLay Community Clustering algorithm (default options) incorporated in the Cytoscape plug-in clusterMaker v.2 (Boston, MA, USA) to provide an optimized layout and structured visualization for biological analysis [106,107]. Each cluster was submitted to enrichment analysis in DAVID v.6.8 using the UniProt IDs of clustered proteins, and then the outputs of gene ontology biological process (GO-BP) and annotation protein pathways from the Kyoto Encyclopedia of Genes and Genomes (KEGG) database were imported to the Cytoscape plug-in EnrichmentMap (FDR < 0.01) [108].

## 4. Conclusions

The TRPV1 channel has become a potentially relevant target for metabolic intervention, including insulin resistance, obesity, type 2 diabetes, and nonalcoholic fatty liver, due to its presence in a variety of metabolic tissues. Even though recent studies have greatly contributed to the knowledge regarding the participation of TRPV1 in metabolism [5,8,9,11,12], there are few reports in the literature, thus far, describing the involvement of TRP channels in the mechanisms responsible for hepatic glucose regulation. A phenotype of hyperglycemia is described in mice lacking TRPV1 channels; however, the mechanism underlying this event remains an open question. Our study contributes to addressing this gap in the literature as we have established which molecular components are involved with reduced glycogen storage in *Trpv1* KO mice. In the present study, we demonstrate that the lack of TRPV1 channels results in reduced glycogen storage, which is related to increased glycogenolysis but also induces high gluconeogenesis. These alterations were associated with molecular signature changes found in multilayer analysis, from transcriptional modulation of the clock system and metabolic genes to altered proteomes in the absence of TRPV1 channels. We suggest that the increased FAO observed in the liver of *Trpv1* KO mice is required to support high hepatic gluconeogenesis since lipid export and malonyl-CoA seem to be reduced. In addition, we found that *Per1* and *Bmal1* genes, two important components of the molecular machinery responsible for circadian regulation of hepatic glucose metabolism, were altered in the liver of *Trpv1* KO mice. Taken together, we demonstrate that pharmacological intervention of signaling pathways, in which the TRPV1 channel is a pivotal component, may modulate glucose metabolism and, therefore, improve liver function. Thus, our results open new avenues to investigate the role of the TRPV1 channel as a key component of hepatic glucose metabolism.

## Figures and Tables

**Table 1:** ANOVA parameters related to gene expression in the liver.

	Time F (DFn, DFd) and *p*-Value		Genotype F (DFn, DFd) and *p*-Value		Interaction F (DFn, DFd) and *p*-Value	
*Akt*	(1, 14) = 3.787	*p* = 0.0720	(1, 14) = 2.713	*p* = 0.1218	(1, 14) = 8.441	*p* = 0.0115 *
*Bmal*	(1, 13) = 34.92	*p* < 0.0001 *	(1, 13) = 6.57	*p* = 0.0236 *	(1, 12) = 0.318	*p* = 0.5824
*G6pase*	(1, 14) = 14.95	*p* = 0.0017 *	(1, 14) = 0.4033	*p* = 0.5356	(1, 14) = 0.1015	*p* = 0.7547
*Glut2*	(1, 14) = 1.429	*p* = 0.2518	(1, 14) = 6.557	*p* = 0.0226 *	(1, 14) = 6.231	*p* = 0.0257 *
*Gsk3b*	(1, 13) = 0.00001	*p* = 0.9970	(1, 13) = 5.553	*p* = 0.0348 *	(1, 13) = 3.349	*p* = 0.0903
*Pepck*	(1, 14) = 2.574	*p* = 0.1310	(1, 14) = 7.956	*p* = 0.0136 *	(1, 14) = 4.875	0.0444 *
*Per1*	(1, 13) = 0.7069	*p* = 0.4157	(1, 13) = 12.65	*p* = 0.0035 *	(1, 13) = 0.2005	*p* = 0.6617
*Pparα*	(1, 15) = 4.982	*p* = 0.0413 *	(1, 15) = 6.201	*p* = 0.0250 *	(1, 15) = 7.609	*p* = 0.0146 *
*Pparγ*	(1, 15) = 3.433	*p* = 0.0837	(1, 15) = 10.9	*p* = 0.0048 *	(1, 15) = 6.232	*p* = 0.0247 *

* Represents statistically significant differences. DF = degree of freedom. DFn is calculated as the total group number—1 and DFd as the total number of mice—1 of each group. F distribution is based on a ratio of DFn and DFd.

**Table 2:** List of 11 exclusively identified kinases, phosphatases, and associated regulator proteins reported to be upstream effectors in each proteome and their correspondingly mapped targets, followed by downstream signaling involved in glycogenesis and glycogenolysis.

Proteins Identified in the Proteomes					Downstream Target Mapped				
Genotype	Upstream Effector	Class	Research Source	Function	Target	Class	Research Source	Function	Downstream Signaling
*Trpv1* KO	DUSP3	Phosphatase	map04010	Inhibition	MAPK14	Kinase	PSP_454221	Inhibition	GSK3B
*Trpv1* KO	MAPK14	Kinase	PSP_454221	Inhibition	GSK3B	Kinase	map04910	Inhibition	GYS2
*Trpv1* KO	MAPK14	Kinase	map04068	Activation	FOXO	TF	map04910	Activation	PEPCK, G6PC1, FBP1, FBP2
*Trpv1* KO	PTPA	Regulator	Guo et al., 2014	Activation	PP2A complex	Phosphatase	Hernández et al., 2010; Yadav et al., 2017	Activation, Activation, Inhibition	GSK3B, FOXO, AMPK
*Trpv1* KO	ENSA	Regulator	Thapa et al., 2020	Inhibition	PP2A complex	Phosphatase	Hernández et al., 2010; Yadav et al., 2017	Activation, Activation, Inhibition	GSK3B, FOXO, AMPK
*Trpv1* KO	PRRC1	Regulator	Kotani et al., 2009	Activation	PKA	Kinase	PSP_449111, map04922, Ahn et al., 2007	Inhibition, Inhibition, Activation, Activation	GSK3B, GYS2, PHKB, PP2A complex
*Trpv1* KO	ARFGEF2	Regulator	Li et al., 2003	Activation	PKA	Kinase	PSP_449111, map04922, Ahn et al., 2007	Inhibition, Inhibition, Activation, Activation	GSK3B, GYS2, PHKB, PP2A complex
*Trpv1* KO	SGK2	Kinase	map04068	Inhibition	FOXO	Transcription factor	map04910	Activation	PEPCK, G6PC1, FBP1, FBP2
WT	MAPK1	Kinase	map04068	Inhibition	FOXO	Transcription factor	map04910	Activation	PEPCK, G6PC1,FBP1, FBP2
WT	ATM	Kinase	PSP_454221	Inhibition	GSK3B	Kinase	map04910	Inhibition	GYS2
WT	PPP2R1B	Phosphatase	map04152	Inhibition	AMPK	Kinase	map04152	Inhibition	GYS2
WT	PPP1CB	Phosphatase	PSP_448786	Inhibition	AMPK	Kinase	map04152	Inhibition	GYS2
WT	PPP1CB	Phosphatase	map04910	Inhibition	PHKB	Kinase	map04922	Activation	PYGL
WT	PPP1CB	Phosphatase	map04910	Activation	GYS2	Kinase	map04922	Glycogen synthase	Glycogen

The research source included the KEGG database (ID: map), PhosphoSitePlus (ID: PSP), and literature data.

**Table 3:** Gene access numbers and sequences of primers and probes.

Template (Access Number)	Primers and Probes
*18S rRNA*	Forward: 5′-CGGCTACCACATCCAAGGAA-3′ Reverse: 5′-GCTGGAATTACCGCGGCT-3′
*Akt* (NM_001165894.1)	Forward: 5′-CCGTGTGACCATGAACGAGT-3′ Reverse: 5′-GGTCGTGGGTCTGGAATGAG-3′
*Bmal1* (NM_001243048)	Forward: 5′-AGCTTCTGCACAATCCACAGCAC-3′ Reverse: 5′-TGTCTGGCTCATTGTCTTCGTCCA-3′ Probe:5′/5HEX/AAAGCTGGCCACCCACGAAGATGGG/BHQ_1/-3′
*G6pase* (NM_008061.4)	Forward: 5′-CAGTGGTCGGAGACTGGTTC-3′ Reverse: 5′-TATAGGCACGGAGCTGTTGC-3′
*Glut2* (NM_0311979.2)	Forward: 5′-TGTTGGGGCCATCAACATGA-3′ Reverse: 5′-GGCGAATTTATCCAGCAGCAC-3′
*Gsk3*β (NM_001347232.1)	Forward: 5′-TGGACAAAGGACTCACCAGG-3′ Reverse: 5′-AAGAGTGCAGGTGTGTCTCG-3′
*Pepck* (NM_011044.3)	Forward: 5′-CGATGACATCGCCTGGATGA-3′ Reverse: 5′-TCTTGCCCTTGTGTTCTGCA-3′
*Per1* (NM_0011065.3)	Forward: 5′-AGCAGGTTCAGGCTAACCAGGAAT-3′ Reverse: 5′-AGGTGTCCTGGTTTCGAAGTGTGT-3′ Probe:5’/5FAM/AGCTTGTGCCATGGACTGTCTACT/BHQ_1/-3′
*Pparα* (NM_011144.6)	Forward: 5′-ACGTTTGTGGCTGGTCAAGT-3′ Reverse: 5′-TGGAGAGAGGGTGTCTGTGAT-3′
*Pparγ* (NM_0011273302)	Forward: 5′-TGTGGGGATAAAGCATCAGGC-3′ Reverse: 5′-CCGGCAGTTAAGATCACACCTAT-3′
*Trpv1* (NM_1001445.1)	Forward: 5′-CAGAGACCTGTGTCGGTTTATG-3′ Reverse: 5′-CATGTTGAGCAGGAGGATGTAG-3′

## Data Availability

The raw MS data associated with this manuscript have been submitted to a public repository (the Mass Spectrometry Interactive Virtual Environment–MassIVE, http://massive.ucsd.edu, accessed on 19 June 2022) and deposited to the ProteomeXchange Consortium (http://www.proteomexchange.org/, accessed on 19 June 2022). Both accessed on 21 June 2022. These data are associated with the identifier MassIVE ID MSV000089696 and Proteome Exchange ID PXD034801. Further information on research design is linked to this article. The authors declare that all other data supporting the findings of this study are available within the article and its supplementary information files.

## Supplementary Materials

The following supporting information can be downloaded at: https://www.mdpi.com/article/10.3390/ijms23137014/s1.
